# 

**Published:** 1885-02

**Authors:** 


### CONTENTS.

ORIGINAL COMMUNICATIONS—

Diagnostic Value of the Soft Palate, as compared with the Tongue in certain Pathological Conditions. By Wm. Abram Love, M. D., Atlanta, Ga. . . . . . . . . . . . . . . . 641

Tobacco Poisoning. By L. G. Hardman, Harmony Grove, Ga. . . 648

SOCIETY REPORTS—

Macon Medical Society. . . . . . . . . . . . . . . . . . 651

Atlanta Medical and Surgical Union. . . . . . . . . . . . 657

CLINIC REPORTS—

Chronic Dislocations.—Successful reduction of a dislocation of the elbow, both bones backwards, of ten weeks standing.—A Clinical Lecture. By W. F. Westmoreland, M. D., Atlanta, Ga. . . . . 665

CORRESPONDENCE—

Our New York Letter.—Asiatic Cholera—New York Orthopœdic Society—Charges against Dr. Fordyce Barker, etc. By Hunter P. Cooper, M. D., New York . . . . . . . . . . . . . . . 668

Letter from Augusta. By J. M. Hull, M. D., Augusta, Ga. . . . 672

BOOK REVIEWS—

Transactions of the Medical Association of Pennsylvania, 1884 674

The Basic Pathology and Specific Treatment of Diphtheria. By Geo. J. Zeigler, M. D. . . . . . . . . . . . . . . . . 686

A Text Book of Hygiene. By Geo. H. Rhoe, M. D. . . . . . . . 687

The Story of My Life. By J. Marion Sims. . . . . . . . . . 688

The International Encyclopædia of Surgery. Edited by John Ashhurst Jr., M. D., . . . . . . . . . . . . . . . . . . . . 688

Books and Pamphlets Received. . . . . . . . . . . . . . . 690

EDITORIAL—

Verdict of a Coroner's Jury . . . . . . . . . . . . . . 693

Medical Association of Georgia . . . . . . . . . . . . . 695

Operation for the Relief of Obstruction of the Gall Duct . . 696

Items . . . . . . . . . . . . . . . . . . . . . . . . 699

Our Advertisers . . . . . . . . . . . . . . . . . . . . 702

